# Editorial: The science of flywheel training: exercise physiology and practical applications

**DOI:** 10.3389/fphys.2023.1241529

**Published:** 2023-06-26

**Authors:** Marco Beato, Javier Raya-González, Jose’ Luis Hernandez-Davo, Sergio Maroto-Izquierdo

**Affiliations:** ^1^ School of Health and Sports Sciences, University of Suffolk, Ipswich, United Kingdom; ^2^ Faculty of Sport Sciences, University of Extremadura, Cáceres, Spain; ^3^ Faculty of Health Sciences, Isabel I University, Burgos, Spain; ^4^ i+HeALTH, European University Miguel Cervantes, Valladolid, Spain

**Keywords:** training, sports, gym, conditioning, strength

## 1 Introduction

Flywheel resistance training was initially designed and implemented to mitigate the negative effects in the absence of gravity on astronauts during space travels (Alkner and Tesch, 2004; Norrbrand et al., 2008). Later on, sport scientists and practitioners understood the advantages offered by this methodology and they started to use flywheel resistance training for several purposes, such as performance development of athletes, injury prevention and clinical rehabilitation (Tesch et al., 2017; Beato and Dello Iacono, 2020; Beato et al., 2021a). Flywheel resistance training offers some specific and unique advantages compared to traditional resistance training, which has increased the popularity of this training methodology. In detail, flywheel training is characterized by the use of rotational inertia to generate resistance throughout the entire range of motion. During the concentric phase, the force applied causes the connected cord/strap to unwind, resulting in the rotation and energy storage of the flywheel–the kinetic energy increases with rotational speed. Upon completing the concentric phase, the cord/strap rewinds, and the user must resist the pull of the flywheel through a braking eccentric phase. By employing the appropriate technique, selecting the right exercise, and using an adequate momentum of inertia, users can achieve an eccentric overload in terms of force and power values (Maroto-Izquierdo et al., 2017; Maroto-Izquierdo et al., 2022). It is important to highlight that the mechanical load performed during both concentric and eccentric phases is the mechanism by which neural and morphological adaptations happen (de Hoyo et al., 2015; Maroto-Izquierdo et al., 2017; De Keijzer et al., 2022). This statement is supported by recent research which reports that flywheel training is a valid method leading to positive morphological changes of muscle structure and architecture, hypertrophy, and strength gains (Maroto-Izquierdo et al., 2017; Allen et al., 2021). Although in the last decade, several researchers have studied the physiological effects of flywheel training and the related acute and chronic adaptations, much research is still needed on this specific argument (Norrbrand et al., 2010; Nuñez and Sáez de Villarreal, 2017; Beato et al., 2020).

Research that clarifies the state-of-the-art technology can be decisive for the optimal implementation of flywheel resistance exercises into training protocols, which is currently limited compared to traditional resistance training (Beato et al., 2019; Allen et al., 2021; de Keijzer et al., 2022b). Therefore, more research is needed to add information and knowledge about its physiological mechanisms as well as its utilization in ecological contexts, which could support the spread of this training modality in applied settings. Frontiers in Physiology recognize the importance of this Research Topic, which is titled “The Science of Flywheel Training: Exercise Physiology and Practical Applications”. This Research Topic aimed to better understand the motivations of some adaptive responses and to elucidate how to better implement such technology into the field of strength training.

## 2 Articles

This Research Topic, “The Science of Flywheel Training: Exercise Physiology and Practical Applications”, contains seven original manuscripts that met the editorial criteria, including three original research articles (Martín-Rivera et al.; Weng et al.; Maroto-Izquierdo et al.), two opinion piece articles (Beato et al.; Maroto-Izquierdo et al.), and two brief research reports (de Keijzer et al.; de Keijzer et al.).

Martín-Rivera et al. studied if concentric linear velocity measurement was a valid method to quantify load and individualize the prescription of flywheel training (Martín-Rivera et al.). Specifically, the authors investigated the relationship between inertial load and mean concentric linear velocity during the flywheel squat exercise in a wide spectrum of moments of inertia. In addition, they compared mean concentric linear velocity and subjective rating of perceived exertion after each load. This article reported that the monitoring of mean concentric linear velocity can be proposed as a valid method to quantify load and to individualize the prescription of flywheel training. Moreover, rating of perceived exertion was a valid and reliable alternative to control flywheel training.

Weng et al. studied the effect of accentuated eccentric loading during flywheel training on the running economy of young male well-trained distance runners (Weng et al.). The authors found that, firstly, this training program improved the muscles’ explosive strength and other neuromuscular functions, as well as improved the athlete’s running economy under 65%, 75%, and 85% peak oxygen consumption (VO_2_peak), which can potentially increase endurance performance; secondly, flywheel resistance training can improve the height, rate of force development, the eccentric utilization ratio in jumps, and other lower limb elastic potential energy indicators of young male well-trained distance runners.

Maroto-Izquierdo et al. investigated the effects of submaximal and supramaximal accentuated eccentric training on changes in lean mass, anabolic hormonal responses, and muscle function (Maroto-Izquierdo et al.). The authors recruited a sample of physically active university students (n = 27), who were randomly assigned to two training groups. Participants performed isotonic training for 10 weeks, with eccentric loads set at 90% (submaximal load) or 120% (supramaximal load) of the concentric one-repetition maximum (1-RM), and a concentric load of 30% of the 1-RM. Supramaximal training was found to be more favorable than submaximal training for increasing 1-RM (effect size = 0.77). However, similar effects on maximal isometric voluntary contraction, local muscle endurance, mechanical power, lean mass, and anabolic hormonal responses (IGF-1 and IL6) were demonstrated after 10 weeks of training with both submaximal and supramaximal eccentric loads. Therefore, although supramaximal loading might be superior for increasing 1-RM, the use of this approach does not appear to be necessary in healthy active individuals.

Beato et al. published an opinion article with the aim to provide methodological bases for the periodization in team sports to practitioners. This paper was structured into four sections: 1) rationale and benefits of flywheel exercise; 2) strength training periodization in team sports; 3) flywheel training periodization in teams ports; and 4) limitations and future directions of flywheel training periodization (Beato et al.).

Maroto-Izquierdo et al. authored an opinion article that aimed to provide the methodological bases for load quantification and testing using flywheel devices in sports. This paper was structured into three sections: 1) load quantification during flywheel exercise; 2) the use of flywheel devices for testing; and 3) limitations and future directions of load monitoring and testing using flywheel devices. (Maroto-Izquierdo et al.), de Keijzer et al., wrote a brief research report that investigated, firstly, the effect of different flywheel moment of inertia on concentric and eccentric peak power and eccentric:concentric peak power ratio during unilateral flywheel leg curl and hip extension exercises. Secondly, the inter-session reliability of peak power in both exercises (de Keijzer et al.). These authors found that a variety of moments of inertia can elicit high eccentric knee flexor demands during unilateral leg curls, whereas higher moments of inertia are needed to achieve an eccentric-overload in peak power during hip extensions. Different exercises may have different inertia-power relationships. Finally, concentric and eccentric peak power measures can be used by practitioners to inform training, while the eccentric: concentric ratio should not be used because of its low reliability.

de Keijzer et al. wrote another brief research report that described the current application and perception of flywheel resistance training amongst therapists working in sport (de Keijzer et al.). The authors recorded the opinions of 52 therapists using an electronic questionnaire and they found that, firstly, most therapists either used or intended to use flywheel training with their athletes. Secondly, more than half suggested they were confident flywheel training could enhance strength and muscular pre-habilitation outcomes. Thirdly, it appeared that therapists would mostly include flywheel training within pre-habilitation or during the later stages of rehabilitation. Although therapists would prescribe most exercises, the squat, rotational exercise, and unilateral leg curl would be the most selected. Finally, the authors reported that the biggest perceived barriers to flywheel training implementation were equipment cost/space, scientific evidence, and scheduling.

Collectively, these studies contribute to a better understanding of flywheel resistance training, including valuable information about quantification, individualization, effects on performance and muscle function, and its potential application in sport and rehabilitation settings.

## 3 Final considerations

This Research Topic aimed to better understand the motivations of some adaptive responses and to elucidate how to better implement such technology into the field of strength training.

The seven articles included gave evidence and opinions around training load monitoring, periodization, exercise selection, and so on, which may facilitate the implementation of flywheel resistance technologies into research and applied settings, may improve the coaches’ practice, and may foster new research ideas.
